# Long-Term Pharmacokinetics of Dalbavancin in ABSSSI and Osteoarticular Settings: A Real-Life Outpatient Context

**DOI:** 10.3390/biomedicines9101288

**Published:** 2021-09-22

**Authors:** Amedeo De Nicolò, Giacomo Stroffolini, Miriam Antonucci, Jacopo Mula, Elisa Delia De Vivo, Jessica Cusato, Alice Palermiti, Giuseppe Cariti, Giovanni Di Perri, Silvia Corcione, Francesco Giuseppe De Rosa, Antonio D’Avolio

**Affiliations:** 1 Laboratory of Clinical Pharmacology and Pharmacogenetics, Department of Medical Sciences, University of Turin, 10149 Torino, Italy; miriam.antonucci20@gmail.com (M.A.); jacopo.mula@unito.it (J.M.); elisa.devivo59@gmail.com (E.D.D.V.); jessica.cusato@unito.it (J.C.); alice.palermiti@unito.it (A.P.); 2 Unit of Infectious Diseases, Department of Medical Sciences, University of Turin, 10124 Torino, Italy; giacomo.stroffolini@gmail.com (G.S.); gcariti@hotmail.com (G.C.); giovanni.diperri@unito.it (G.D.P.); silvia.corcione@unito.it (S.C.); francescogiuseppe.derosa@unito.it (F.G.D.R.)

**Keywords:** pharmacokinetics, antibiotics, anti-infective, quantitative pharmacology, pharmacokinetics-pharmacodynamics

## Abstract

Dalbavancin is a lipoglycopeptide approved for treatment of Gram-positive infections of skin and skin-associated structures (ABSSSI). Currently, off-label use at high dosages for osteoarticular infections deserves attention. This work aimed to study the long-term plasma pharmacokinetics of dalbavancin in outpatients with ABSSSI or osteoarticular infections, treated either with one or two 1500 mg doses of dalbavancin. A liquid chromatography-tandem mass spectrometry method was used to measure total dalbavancin concentrations in plasma samples. The results were analyzed through a non-compartmental analysis (NCA). Breakpoint minimum inhibitory concentration (MIC) was used to calculate AUC/MIC and T > MIC parameters, adjusted by 93% protein binding. A total of 14 patients were enrolled, 11 with osteoarticular infection and 3 with ABSSSI. Long-term pharmacokinetics showed median T > MIC (0.125 mg/L) of 11.9 and 13.7 weeks for single and dual dose, respectively. Similarly, median AUC_0-2w_/MIC ratios of 20,590 and 31,366 were observed for single and dual dose regimens, respectively. No adverse events were observed, and treatment success was achieved in 12/14 patients. Failure was associated with the worst clinical conditions, bone infections, and single dose. The results of this study show that dalbavancin exposure exceeds previously suggested pharmacodynamic targets. Optimization of these targets is needed for the osteoarticular setting.

## 1. Introduction

Dalbavancin (DBV) is a recently introduced semi-synthetic second-generation lipoglycopeptidic antibiotic drug, effective for the treatment of infections caused by gram-positive bacteria, particularly against streptococci and staphylococci [1,2,3,4,5].

Compared to its precursor teicoplanin, the molecular modifications allowed to obtain higher lipophilicity, increasing its half-life (t1/2, around 9 days), also by means of a strong reduction of its renal clearance [6,7,8]. This is also due to its high percentage of binding to plasma proteins (mainly albumin), which was described to vary from nearly 98% in rats to 93% in humans [6,9,10]. The long t1/2 allows its use for the treatment of acute bacterial skin and skin-structure infections (ABSSSIs) with two possible approved dosing regimens: a first loading dose of 1000 mg followed by a second 500 mg dose after 7 days or a single dose of 1500 mg [1,11,12,13]. Recently, the convenient PK/PD profile of this drug, as well as the capability to spread through many tissues (e.g., synovia, blister fluid, bone, and bone marrow), and its effect on biofilm increased the interest in its use for other “off-label” indications [13,14,15,16], such as for the treatment of joints, intravascular and surgical site infections, and for osteomyelitis and endocarditis [7,14,15,16,17,18,19,20,21,22]. In this scenario, several works reported encouraging results for these alternative indications [13,15,16,23]. Nevertheless, the currently approved posology for ABSSSIs may be suboptimal for the treatment of osteoarticular infections, considering that DBV concentrations in bone were described as comparable to the free fraction in plasma [6,7,8,10,24].

Considering the high tolerability, an alternative off-label 1500 mg dual dose regimen has been suggested as more convenient and effective in several reports [14,15,17,23]. Nevertheless, few PK data are available about this off-label high dose regimen in real-life clinical use, since the vast majority of PK data were derived from modeling [8,25] and because the length or choice of therapy was not evidence-driven or relying on clear PK data. Moreover, previous PK studies conducted on volunteers investigated DBV concentrations in the first 2 months [8,10,26]. Therefore, poor knowledge is currently available on long-term PK in real-life settings, particularly beyond 2 months. Currently, also considering that the COVID-19 pandemic has caused an extreme need to reduce the time of hospitalization, long-acting antibiotics such as DBV should also be evaluated by the means of prolonged effectiveness, favoring the management shift from inpatient to outpatient services [25]. Therefore, data about long-term PK for DBV administered at high dosages, particularly for the treatment of bone-associated infections, may be especially helpful today.

In this work, we aimed to describe the long-term PK profiles in a real-life clinical context of outpatients with ABSSSI or bone and joint infections, treated with one or two 1500 mg doses of DBV (one week apart). As a secondary endpoint, we aimed at describing theoretical PK/PD parameters in this cohort, by comparing the observed PK data with the EUCAST breakpoint MIC values for DBV-susceptible strains.

## 2. Materials and Methods

### 2.1. Patients’ Enrolment and Treatment

Patients with Gram-positive infections, with previous therapeutic failures to other antibiotic regimens or need for simplification and eligible for treatment with DBV were enrolled in the “Appropriatezza Farmacologica della Terapia Anti-Infettiva” (ethical approval n. 0,040,388 23/04/2020) clinical study.

Patients stopped all previous antibiotic therapies and were selected for a single 1500 mg or dual 1500 mg DBV infusion (1500 mg × 2, one week apart) based on guidelines, investigator judgment and emerging data from the literature [13,15,16,23]. In our institution, we recommend DBV for ABSSSIs (as per approved indication) or other Gram-positive infections with a specific off-label request within our study protocol, based on recent studies and theoretical PK/PD considerations, as no other indication has been approved up to date and no conclusive data exist; we recommend two doses one week apart mainly in the setting of osteoarticular infections [13,15,16,23]. Drug administration was performed intravenously in 30 min.

Patients with compatible conditions were discharged from hospital either after the 1st or 2nd administration, from which time they were treated as outpatients. Monitoring for eventual adverse events (AE) and effectiveness was performed via weekly periodical visits including clinical and laboratory examination. When not applicable or due to the COVID-19 emergency, visits were carried out via telemedicine. No patient had viral co-infections. Clinical, microbiological, and demographic data were collected for each patient at the time of enrolment. Microorganisms were isolated from either blood culture, swab, or surgical samples, and the antibiogram was performed using ETEST. No susceptibility testing was available for the direct determination of DBV MIC; therefore, surrogate categorization of susceptibility was performed based on the MIC for vancomycin, as previously reported and indicated by EUCAST and by Jones et al. [27]. Therefore, susceptibility to vancomycin (minimal inhibitory concentration, MIC ≤ 2 mg/L) was interpreted as DBV MIC ≤ 0.125 mg/L (nearly 97% probability from Jones et al.), the EUCAST suggested breakpoint for staphylococci and streptococci. The cure was defined as resolution of symptoms, microbiological cure, or no evidence of pathology as assessed by radiology techniques when applicable. Body surface area (BSA) was estimated by the Du Bois formula.

### 2.2. PK Evaluation

Blood sampling in 7 mL lithium/heparin tubes was scheduled, after each DBV dose, at the following times: 0, 0.5 h (end-of infusion), 1 h, 1 week, 2 weeks, 3 weeks, 1 month, and then every two weeks, for the quantification of DBV. Deviations in the sampling schedule were allowed for timings later than 4 weeks after the last administration, based on outpatients’ exigencies and considering the management issues related to the COVID-19 outbreak. The calculation of the terminal half-life was performed only for patients who completed at least 8 weeks of PK follow-up. Blood samples underwent centrifugation at 1400× *g* at 4 °C to obtain plasma, which was stored at −80 °C before analysis (max 1 month). The quantification of DBV was performed through a validated UHPLC-MS/MS analytical kit (Kit-System^®^ Antibiotics, kindly donated by CoQua Lab, Turin, Italy) showing acceptable accuracy and precision (bias and coefficient of variation < 15%) according to EMA and FDA guidelines [28,29] and a LLOQ value of 0.3 mg/mL.

Briefly, the analytical process consisted of a fast protein precipitation protocol of 50 µL of plasma with 150 µL precipitation solution, centrifugation at 10,000× *g*, to obtain high and reproducible recovery of the analyte (mean 104% and CV 8.1%), 1:10 dilution with aqueous solution, and analysis. The chromatographic separation was obtained with a binary gradient run of 10 min with two mobile phases and a reverse-phase UHPLC column (Kit-System antibiotics analytical column, CoQua Lab, Turin, Italy). Eventual variability in recovery or matrix effect were adjusted by the use of a Stable-Isotope Linked Internal Standard (SIL-IS, 2H6-DBV; Alsachim, France) [30]. The quantification traces for DBV and SIL-IS were 908.9 > 730.4 and 912.4 > 733.9, respectively.

**Figure 1:**
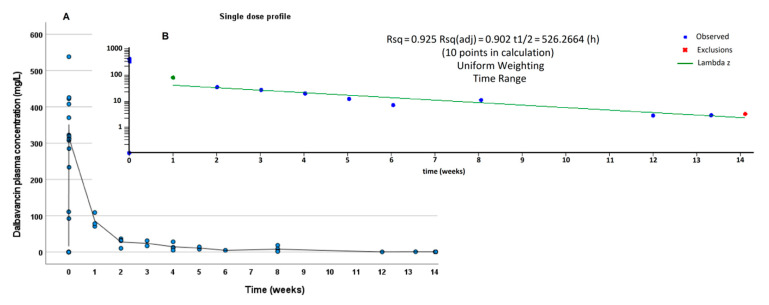
Summary of the mean PK data deriving from a single 1500 mg infusion of DBV (pooled in the first week) in linear-linear scale (**A**) and in log-linear scale (**B**). The blue line indicates the time range for the estimation of terminal λz.

**Figure 2:**
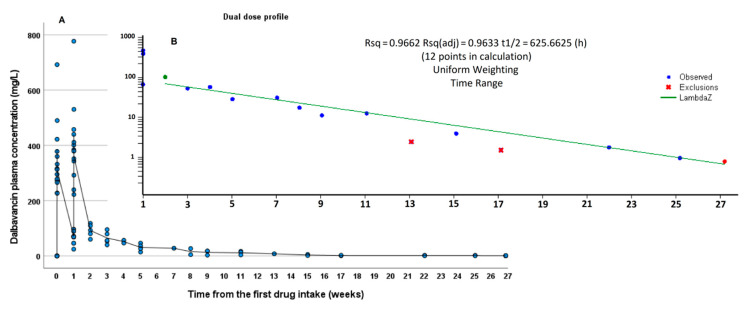
Summary of the mean DBV PK data from the full PK follow-up for the dual 1500 mg infusion group (4 patients) in linear-linear scale (**A**) and in log-linear scale (**B**, limited to the second dose administration). The blue line indicates the time range for the estimation of terminal λz.

PK calculations, including, AUC (area under the curve)_0-1w_, AUC_0-2w_, extrapolated AUC_0-∞_, and t ½ were performed through Phoenix WinNonlin software (Ver. 8.1, Certara, Princeton, NJ, USA). Mean PK non-compartmental analysis (NCA), reported in Figure 1 and Figure 2, was performed following a trapezoidal “linear-up/log-down” model with IV infusion. The mean concentration data corresponding to the single dose group and to the first dose of dual dose group (for the first week) were used to describe the mean PK profile of a single 1500 mg DBV dose; similarly, the mean PK NCA of the dual dose group was performed on the data from the first and second dose of the dual dose group: total AUCs for the dual dose group were calculated as the sum of the first dose AUC_0-1w_with the corresponding AUC parameters from the second dose.

The terminal t1/2 was evaluated in the period from 1 week to the last available timing. The time ranges for the calculation of λz (and t1/2) and eventual exclusions were defined based on reaching coefficients of determination (R^2^), absolute and adjusted, higher than 0.90 and 0.8, respectively.

Then, NCA was also independently repeated with data from each single patient: the determination of terminal t1/2 and AUC_0-∞_ were performed only for patients who completed at least 2 months of follow-up.

### 2.3. Pharmacodynamic Evaluation

Considering the low availability of susceptibility testing for DBV in the clinical practice, after determination of patients’ strains susceptibility to vancomycin (MIC ≤ 2 mg/L), the observed PK data were compared with the EUCAST determined breakpoint MIC of 0.125 mg/L in order to calculate the pharmacokinetic/pharmacodynamic (PK/PD) parameters assuming DBV-susceptible strains. Further analysis was conducted considering a 0.250 mg/L MIC (general breakpoint in case of empirical therapy) in order to describe the PK/PD parameters in the worst hypothetical clinical context. AUC_0-1w_/MIC and, particularly, AUC_0-2w_/MIC were previously described as the most important predictors of treatment efficacy and, therefore, these were included in this analysis [7]. T > MIC was calculated based on the terminal t1/2 for each patient as a possible marker of the long-term prophylactic effect. Each calculation was performed adjusting the MIC value by considering the theoretical free fraction of DBV: freeDBV = totalDBV/(1-boundDBV). The literature-derived protein binding percentage of 93% (0.93) was considered for this calculation [1,6,7].

### 2.4. Statistical Analysis

All statistical analyses were performed through Excel and SPSS 26.0 (IBM, Armonk, NY, USA). Descriptive data are reported as median and interquartile ranges (IQR). Correlations between continuous data were evaluated through Pearson’s correlation tests. Differences between groups have been tested through the Mann–Whitney non-parametric rank test.

## 3. Results

### 3.1. Patients’ Characteristics

Fourteen outpatients treated with either single or dual 1500 mg dose of DBV were enrolled in this study. The overall clinical characteristics of these patients are summarized in Table 1, while median anthropometric and demographic characteristics were as follows: age of 62 years old (IQR 54–74), height of 1.80 m (IQR 1.74–1.82), weight of 75.0 kg (IQR 68.0–89.2), BMI of 24.6 kg/m^2^ (IQR 22.3–26.3), and BSA of 1.95 (IQR 1.84–2.10).

Eleven out of fourteen patients had bone-associated infections (79%), while others had ABSSSIs. Microorganisms, when isolated, were oxacillin-resistant in 57% (8/14) of cases; no vancomycin-resistant strains were isolated, indirectly confirming theoretical DBV-susceptibility. In two cases, the isolation of the pathogen was not possible due to the nature of the infection, technical impossibility, or inoperability of the patient. Reasons for treating bone infections with DBV were simplification (4/11) or failure (7/11) to previous regimens. A single 1500 mg dose was administered to six patients, three with ABSSSIs, two with septic arthritis, and one with chronic osteomyelitis. A dual 1500 mg dose administration was given to eight patients: two with spondylodiscitis, three with chronic osteomyelitis, one with a prosthetic infection, one with septic arthritis, and one with concomitant septic arthritis and spondylodiscitis. The median age was 60 years (IQR 52–75) and 62 (IQR 44–76) in the single and dual dose groups, respectively. Median weight, body mass index (BMI), and BSA were 85 kg (IQR 66–83), 27.0 kg/m^2^ (IQR 21.6–26.7), and 1.89 m^2^ (IQR 1.86–2.00) in the single dose group and 70 kg (IQR 61–87), 22.4 kg/m^2^ (IQR 21.6–26.0), and 1.89 m^2^ (IQR 1.76–2.09) in the dual dose group, respectively. The median estimated glomerular filtration rate (eGFR) was 81 (IQR 66–111) in the single dose group and 102 (IQR 72–138) in the dual dose group. Six patients had, according to eGFR, chronic kidney disease (CKD) stage II and one patient CKD stage III. No significant differences were found between the two dosing groups.

### 3.2. PK Results

DBV concentrations were detectable (>0.3 mg/L) at all time points, up to 8 months from the last administration. Mean PK calculations were separately applied to the single and dual dose groups and were summarized in Table 2. DBV showed a multiphasic PK profile, showing a relatively fast decline immediately after the infusion and a progressive increase in the t1/2, reaching an extremely long terminal t1/2 during the second week after the infusion, ranging from 526 h in the single dose group to 626 h in the dual dose group (Figure 1 and Figure 2, respectively). The R^2^ values for the determination of terminal t1/2 were 0.925 and 0.966, respectively, confirming good reliability of the calculation.

The overall mean PK data were summarized in Table 2, the total observed AUC_0-1w_, AUC_0-2w_, and AUC_0-∞_ were, respectively, 27,230 h × mg/L, 35,647 h × mg/L, and 54,666 h × mg/L for the single dose; for the dual dose group, the overall AUC_0-1w_, AUC_0-2w_, and AUC_0-∞_ were 25,110 h × mg/L (corresponding to the first dose AUC_0-1w_), 58,012 h × mg/L (sum of the first and second dose AUC_0-1w_), and 116,196 h × mg/L for the dual dose. The percentage of extrapolated AUC_0-∞_ (%AUC_ext_) was <10% both in the single and dual dose groups.

Patient-specific PK parameters are reported in Table 3. Terminal t1/2 and AUC_0-∞_ was estimable in 11 patients, who completed at least 2 months of PK follow-up. Maximum concentrations of DBV were inversely correlated with patients’ height both for the first and second dose (R = −0.643, *p* < 0.01 and R = −0.914, *p* = 0.001, respectively).

**Figure 3:**
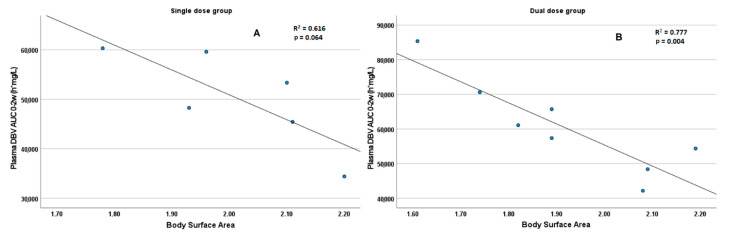
Scatter plots summarizing the distribution of DBV overall AUC_0-2w_values in single dose group (**A** panel) and in the dual dose group (**B** panel), based on the BSA (m^2^). Borderline significance was observed for the single dose group, while the correlation was highly significant in the dual dose group.

Similarly, BSA was inversely correlated with the total AUC during the first 2 weeks of treatment in the dual dose group (R = −0.881, *p* = 0.004) and with borderline significance in the single dose group (R = −0.785, *p* = 0.064), as depicted in Figure 3.

### 3.3. PK-PD Evaluation

The observed PK parameters were compared with the PD susceptibility breakpoints indicated by EUCAST and are summarized in Table 4.

Considering the breakpoint MIC for staphylococci and streptococci (0.125 mg/L) and the effect of 93% plasma protein binding, mean PK results showed cumulative AUC_0-1w_/MIC, AUC_0-2w_/MIC, and AUC_0-∞_/MIC ratios of 16,246, 19,959, and 30,608 for the single dose and 18,422, 32,481, and 65,059 for the dual dose, respectively. Patients’ specific AUC/MIC data are summarized in Table 4: all the AUC_0-2w_/MIC values surpassed a previously suggested cut-off of 1000 (for staphylococci) [7] of at least 8 folds, even considering the highest breakpoint MIC. Taking into account the 0.125 mg/L breakpoint MIC, the median T > MIC values from the two groups ranged from 11.9 w (IQR 9.3–16.7) to 13.7 w (IQR 12.6–18.8) for the single and dual dose groups, respectively; these values changed to 9.0 w (IQR 6.5–13.4) and 9.7 w (IQR 8.2–14.7) if the general breakpoint of 0.250 mg/L was considered.

### 3.4. Clinical Outcome and Safety

**Figure 4:**
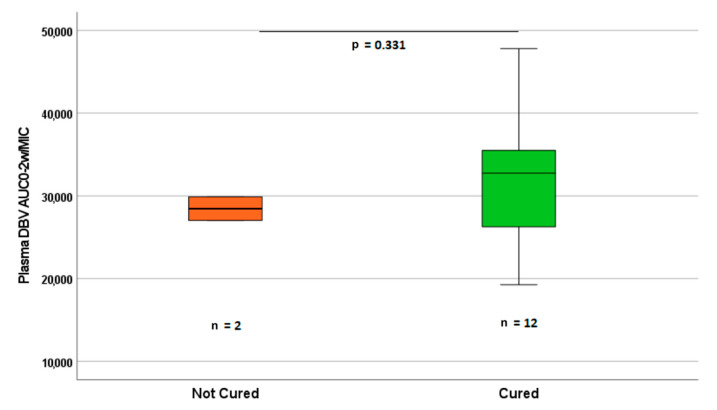
Distribution of the minimum estimated DBV AUC_0-2w_/MIC ratios (based on the breakpoint MIC of 0.125 mg/L, sensitivity cutoff for staphilococci) for patients who achieved a full cure (*n* = 12) and those who did not (*n* = 2). Sensitivity to DBV (MIC ≤ 0.125) was assessed by indirect testing of sensitivity to vancomycin.

The observed outcomes and adverse events are summarized in Table 1. The majority of patients (86%; 12/14) achieved a complete cure, while two patients had a partial improvement but were not cured at the end of the observation study period. Patients who did not reach a complete cure had AUC_0-2w_/MIC values slightly lower than the median value in the group of patients who were cured, although this difference did was not statistically significant (*p* = 0.331, Figure 4), maybe due to the low sample size and to the lack of DBV specific MIC values. The two cases of treatment failure belonged to the single dose group, had osteoarticular infections, and had generally worst clinical and microbiological conditions (one was diabetic and obese, the other one had a vancomycin MIC of 2 mg/mL). Four patients had undergone surgery before treatment and one after because of uncontrolled infection. Despite the very high and prolonged exposure to DBV, no adverse events were registered throughout the observation period.

## 4. Discussion

In this study, we reported the real-life PK and theoretical PK/PD profiles of DBV both in the single 1500 mg and in the off-label dual 1500 mg regimens. Reasons for treating with DBV were simplification (4/11) or failure of previous antibiotic therapy (7/11); reasons for failure of prior antibiotic therapy were probably type of infection, largely hard to treat, but no further hypothesis can be speculated based on the actual microbiological or PK/PD data. The enrolled patients had either ABSSSIs or bone infections, including septic arthritis, spondylodiscitis, osteomyelitis, and prosthetic infections. Satisfactory treatment success (86%) and absence of adverse events confirmed the appropriateness of DBV in this setting.

PK data confirmed a multiphasic profile, with a gradual increase in the t1/2, reaching values higher than 500 h, probably due to high storage within tissues and protein binding, which can sustain a prolonged recirculation from tissues during the terminal phase. Nevertheless, due to low sample size, further studies are needed to verify this hypothesis. In accordance with the work from Dunne et al. [8], the DBV exposure in the first weeks appeared correlated with patients’ BSA, while no statistically significant correlation was found with patients’ eGFR, probably due to the low sample size. Interestingly, although our observed concentrations and PK profiles were in accordance with several previous reports [6,7,8], the observed concentrations (particularly the Cmax) differed from the ones described and modeled by Cojutti et al. [25]. In our opinion, this is due both to the different infusion duration (0.5 h vs. 2 h) and to a methodological difference in the analytical approach between the two studies. In fact, Cojutti et al. applied a previously reported method [31] based on sample dilution before LC-MS analysis, with minimal use of organic solvents throughout the protocol. This is probably not enough to completely separate DBV from plasma proteins during sample preparation, potentially leading to a systematic underestimation of the total DBV concentration. Conversely, in this work, we applied a validated analytical kit capable of quantifying total DBV concentration in plasma with high accuracy, using a SIL-IS to compensate for potential fluctuations in analyte recovery and matrix effect.

The observed values that AUC_0-2w_/MIC adjusted by 93% protein binding, which was suggested to be the most reliable PK/PD marker for DBV, remained for all patients higher than 17,000 (single dose) and 23,000 (dual dose), considering the breakpoint MIC of 0.125 mg/L. These values greatly exceed the proposed target AUC_0-2w_/MIC of 1000 for staphylococci in ABSSSI [7], even in patients who did not achieve treatment success, suggesting that specific PK/PD targets should be defined in the context of bone infections.

Despite the adjustment of MIC values by 93%, protein binding could not be completely free of errors, particularly considering eventual inter-patient variability, which was not possible to assess in this study. The considered adjusted MIC values in this study are very near to the ones experimentally observed in-vitro in the work from Leighton et al. in presence of human serum (range 1.22–2.04 mg/L vs. 1.71–3.42 mg/L by our calculation) [10]. The evidence of a T > MIC higher than 6 weeks in all patients, even considering the highest breakpoint MIC, suggests a potential prophylactic effect against bacterial dissemination and/or reinfection in particularly frail individuals, in accordance with several previous works [14,18]. It is particularly noteworthy that a 6-week period would fit within the recommended time length for the majority of bone or vascular Gram-positive infections. It remains to be proven if this characteristic could play a role in modifying the microbiome and subsequently lead to any alterations in bacterial flora.

This, together with the high success rate and tolerability, suggests the eligibility of these off-label regimens for early discharge from hospital, reducing the risk of nosocomial infections (particularly important during the COVID-19 pandemic) and the costs for the health system. Interestingly, the two patients who did not reach a complete cure belonged to the single dose group, had bone-associated infections, and had slightly lower AUC_0-2w_/MIC values than patients who achieved therapeutic success (Figure 4). Among these two cases, one was a diabetic, obese female and had a long history of superposed infections complicating her chronic osteomyelitis. She had failed previous regimens targeting her repeatedly isolated methicillin-susceptible *Staphylococcus aureus* (MSSA). A few weeks after DBV administration, after slight improvement, a multidisciplinary team composed of a diabetologist, an orthopedic doctor, and an infectious diseases specialist opted for amputation. In that context, intra-operative culture confirmed MSSA chronic osteomyelitis, excluding the possibility of a Gram-negative superinfection that would have complicated her recovery. The main limitations from this study consist of the lack of intermediate time points for the PK evaluation during the first hours post-infusion (due to the exigencies of outpatients) which could cause a slight overestimation of the early AUC values, the unavailability of specific DBV MIC testing, the relatively low sample size, and the loss of the very late timings for four patients, in all cases due to the COVID-19 emergency. Nevertheless, the observed data resulted in good model fitting for NCA (R^2^ higher than 0.90 for AUC estimation) and were in accordance with the predicted PK from previous studies [7,8]. From a theoretical PK and PK/PD perspective, our data support a potential longer antibiotic activity than previously considered both for single or double 1500 mg dose administration and for a possible mis-dosing of this molecule in different settings. The evidence of treatment failures with the single dose administration for treatment of bone infections, together with the lower AUC_0-2w_/MIC ratio, although not statistically significant, confirms the need for a second dose in this setting [23,25]. This should be taken into account for further studies on wider cohorts and when considering DBV for different indications other than ABSSSI.

To better explain the duration of the antibiotic effect, further studies on larger cohorts, including the direct determination of the free DBV concentration and its PK within tissues, deserve attention.

## Figures and Tables

**Table 1:** Clinical features of the enrolled patients. Vancomycin MIC ≤ 2 was considered as a categorical surrogate of susceptibility to DBV. MSSA: methicillin-susceptible *Staphylococcus aureus*; MRSA: methicillin-resistant *Staphylococcus aureus*; MRSE: methicillin-resistant *Staphylococcus epidermidis*.

N	N. of DBV Doses	Gender	Obesity	eGFR	Diabetes	Indication	Etiology	MIC for Vancom.	Previous Surgery	Previous Antibiotic Therapy	AE	Outcome
1	1	F	yes	86.5	yes	Chronic osteomyelitis	MSSA	≤1	No	Piperacillin-tazobactam + teicoplanin	none	Not Cured
2	1	M	no	107.5	no	Septic arthritis	MRSE	≤1	Yes	Teicoplanin	none	Cured
3	2	M	no	53.4	yes	Spondylodiscitis	MSSA	≤0.5	No	Cefazolin, ceftriaxone, daptomycin	none	Cured
4	2	M	no	139.9	yes	Spondylodiscitis	MRSE	2	No	Teicoplanin	none	Cured
5	2	M	no	113.8	no	Chronic osteomyelitis	MRSA	1	Yes	Teicoplanin	none	Cured
6	1	M	yes	75.0	yes	ABSSSI	-	-	No	Levofloxacin	none	Cured
7	1	F	no	62.2	no	ABSSSI	MRSE	≤2	No	Amoxicillin/clav.	none	Cured
8	1	M	no	121.1	no	Septic arthritis	MRSA	2	Yes	Daptomycin	none	Not Cured
9	1	M	no	69.9	no	ABSSSI	-	-	No	Amoxicillin	none	Cured
10	2	M	no	68.6	no	Prosthetic infection	MRSA	1	Yes	Teicoplanin + rifampin	none	Cured
11	2	M	no	131.0	yes	Chronic osteomyelitis	MRSA	≤1	No	Amoxicillin/clav., Vancomycin, daptomycin, cefazolin	none	Cured
12	2	M	no	82.5	no	Septic arthritis and spondylodiscitis	MRSA	1	Yes	Daptomycin	none	Cured
13	2	M	no	142.0	no	Chronic osteomyelitis	MSSA	≤0.5	No	Ceftriaxone, daptomycin + ceftarolin	none	Cured
14	2	F	no	91.0	no	Septic arthritis	*Streptococcus dysgalactiae*	≤0.5	No	Ceftriaxone	none	Cured

(-) indicates missing data.

**Table 2:** Summary of the mean PK NCA of the last dose in the two treatment groups, single vs. dual 1500 mg intravenous doses, administered through 30 min infusion. Total AUCs for dual dose regimen have been calculated by the sum of the first dose AUC_0-1w_ with the AUC from the second dose (e.g., total AUC_0-2w_ = first dose AUC_0-1w_ + second dose AUC_0-1w_).

PK Parameters	Single Dose	Dual Dose (First Dose)	Dual Dose (Second Dose)
C max (mg/L)	390.1	359.0	431.2
Observed AUC_0-1w_ (h × mg/L)	27,230	25,110	32,902
Observed AUC_0-2w_ (h × mg/L)	35,647	n.a.	45,658
Observed AUC_0-∞_ (h × mg/L)	54,666	n.a.	91,086
Dual dose AUC_0-2w_total (h × mg/L)	n.a.	n.a.	58,012
Dual-dose AUC_0-∞_total (h × mg/L)	n.a.	n.a.	116,196
Terminalt_1/2_ (h)	526	n.a.	626

n.a., not applicable.

**Table 3:** Summary of PK parameters measured for each patient. Total AUCs for dual dose group were calculated as the sum of the first dose AUC_0-1w_with the corresponding second dose AUC.

Pt #	N of Doses	Previous Dose	Last Dose Parameters							Overall Exposure	
		Obs.AUC_0-1w_ (h × mg/L)	LastC_max_ (mg/L)	Obs. AUC_0-1w_ (h × mg/L)	Obs. AUC_0-2w_ (h × mg/L)	Obs. AUC_0-∞_ (h × mg/L)	T_last_ (w)	% ext.AUC	Term.t_1/2_ (h)	TotalAUC_0-2w_ (h × mg/L)	TotalAUC_0-∞_ (h × mg/L)
1	1	n.a.	315.5	27,134	37,539	84,972	14	7.9	860	n.a.	n.a.
2	1	n.a.	307.9	23,059	31,353	41,917	8	25.0	225	n.a.	n.a.
3	2	22,786	384.1	32,426	48,940	107,603	27	1.2	671	55,212	130,389
4	2	24,984	379.5	40,791	60,347	115,735	27	0.8	703	65,775	140,719
5	2	28,150	401.3	32,937	41,579	-	2	-	-	61,087	-
6	1	n.a.	422.4	28,701	39,157	56,314	13	0.5	614	n.a.	n.a.
7	1	n.a.	538.2	39,338	53,086	78,307	14	1.0	441	n.a.	n.a.
8	1	n.a.	322.5	29,196	38,910	-	2	-	-	n.a.	n.a.
9	1	n.a.	407.8	32,585	43,276	-	4	-	-	n.a.	n.a.
10	2	24,543	239.5	24,554	37,039	74,069	25	0.4	640	49,097	98612
11	2	16,705	370.2	25,457	30,090	35,552	17	1.2	310	42,162	52,257
12	2	25,955	457.8	28,414	47,210	68,816	27	1.0	647	53,871	94,273
13	2	28,413	440.4	32,685	42,411	56,544	9	14.2	628	61,098	84,957
14	2	40,035	776.9	45,322	58,713	100,922	25	0.7	463	85,357	140,957

(-) indicates not computable parameters, due to insufficient number of timings; n.a. means not applicable.

**Table 4:** Summary of the PK/PD parameters calculated for each patient. The PK/PD calculations corrected by protein binding were performed using MIC/0.07 as a correction factor, simulating human plasma protein binding. The first dose AUC_0-1w_was considered for the dual dose group. Total AUC_0-2w_ for the dual dose group was calculated as the sum of the first dose AUC_0-1w_ and the second dose AUC_0-1w_.

PT Code	Protein-Binding Corrected PK/PD Parameters								Outcome
	Last DoseCmax/MIC		AUC/MIC				T > MIC (w)		
	MIC = 0.125 mg/L	MIC = 0.250 mg/L	AUC_0-1w_/MIC (First Dose)		AUC_0-2w_/MIC (Total)		MIC = 0.125 mg/L	MIC = 0.250 mg/L	
			MIC = 0.125 mg/L	MIC = 0.250 mg/L	MIC = 0.125 mg/L	MIC = 0.250 mg/L			
1	177	88	15,195	7598	21,022	10,511	24.6	19.5	Not cured
2	172	86	12,913	6457	17,558	8779	7.9	6.6	Cured
3	215	108	12,760	6380	30,919	15,459	20.5	16.6	Cured
4	213	106	13,991	6996	36,834	18,417	20.6	16.4	Cured
5	225	112	15,764	7882	34,209	17,104	-	-	Cured
6	237	118	16,073	8036	21,928	10,964	9.8	6.1	Cured
7	301	151	22,029	11,015	29,728	14,864	14.0	11.4	Cured
8	181	90	16,350	8175	21,790	10,895	-	-	Not cured
9	228	114	18,248	9124	24,235	12,117	-	-	Cured
10	134	67	13,744	6872	27,494	13,747	12.2	9.0	Cured
11	207	104	14,256	7128	23,611	11,805	7.7	6.0	Cured
12	256	178	15,912	7956	30,168	15,084	13.7	9.7	Cured
13	247	124	18,304	9152	34,215	17,107	13.6	7.9	Cured
14	435	218	25,380	12,690	47,800	23,900	18.7	15.8	Cured

(-) indicates missing data due to insufficient PK follow-up.

## Data Availability

Raw data will be provided on request.

## Abbreviations

ABSSSI	acute bacterial skin and skin-structures infections
NCA	non-compartmental analysis
MIC	minimum inhibitory concentration
AUC	area under the concentration-time curve
PK	pharmacokinetics
PD	pharmacodynamics
BSA	body surface area
LLOQ	lower limit of quantification
IS	internal standard
IQR	interquartile range
eGFR	estimated glomerular filtration rate
CKD	chronic kidney disease
LC-MS	liquid chromatography with mass spectrometry
MSSA	methicillin-sensitive *Staphylococcus aureus*
MRSA	methicillin-resistant *Staphylococcus aureus*
MRSE	methicillin-resistant Staphylococcus epidermidis
T > MIC	time range of drug concentrations over the minimum inhibitory concentration
λz	elimination constant
